# Real-world semaglutide in obesity cohort: effectiveness, safety and persistence across sex, BMI, and prior GLP-1RA treatment

**DOI:** 10.3389/fendo.2026.1819337

**Published:** 2026-07-28

**Authors:** Ilaria Milani, Maria Eugenia Parrotta, Marianna Chinucci, Emanuele Franzò, Elena Gintoli, Francesco Franco, Paolo Sbraccia, Valeria Guglielmi, Danila Capoccia

**Affiliations:** 1 Department of Medico-Surgical Sciences and Biotechnologies, Faculty of Pharmacy and Medicine, University of Rome La Sapienza, Lazio, Italy; 2 Department of Systems Medicine, University of Rome Tor Vergata, Rome, Italy; 3 Obesity Medical Center, University Hospital Policlinico Tor Vergata, Rome, Italy

**Keywords:** adherence, effectiveness, GLP-1 receptor agonist, obesity management medications, real-world evidence, sex-differences, switching, weight loss

## Abstract

**Background:**

Semaglutide has shown significant efficacy in several clinical trials for the treatment of obesity. However, real-world data on early weight loss and metabolic response in obesity without diabetes cohort are still limited, particularly with respect to individual patient factors. This study aims to evaluate the real-world effectiveness of semaglutide on anthropometric and metabolic parameters in an obesity cohort, including weight loss (kg and percent) and the percentage of participants achieving the 5%, 10% and 15% weight loss targets at mean 3-month, 6-month and 12-month follow-up. In addition, an assessment of safety and reasons for discontinuation, and sub-analyses of anthropometric outcomes by prior liraglutide exposure, sex, and BMI categories will be included.

**Methods:**

In this retrospective study, data were collected from adults with overweight/obesity without diabetes treated with weekly subcutaneous semaglutide for at least 3 months and analyzed at 2-4 (T1), 5-8 (T2), and 9-15 (T3) months of follow-up.

**Results:**

Among 248 patients (55.0 years, 34.8 kg/m^2^, 77.5% female), only 10.4% and 5.7%, respectively, discontinued due to non-severe gastrointestinal adverse events or cost. Significant weight loss (WL), %WL goal attainment, and improvements in adiposity and metabolic outcomes, including hepatic steatosis, fibrosis, and visceral adiposity indices, were observed at all follow-up visits. Early metabolic improvements occurred regardless of %WL≥5 target. Reduced early anthropometric response in switchers (24%) vs. naive subjects with similar one-year outcomes and no significant differences by sex or baseline BMI category were observed.

**Conclusions:**

These results confirm semaglutide’s effectiveness on cardiometabolic risk in real-world obesity management, with a safety profile and early metabolic effects independent of weight loss. The reduced early response in switchers and at low doses highlights the importance of dose optimization to prevent suboptimal initial outcomes after switching. The lack of differences by sex and BMI category supports further studies stratifying patients by age and metabolic risk rather than BMI alone. Low persistence highlights the need for structured, patient-centered support to improve adherence and a better understanding of the drivers of discontinuation.

## 1 Introduction

Obesity is a growing global health issue characterized by excess body fat (1) and driven by complex biological mechanisms, including hypothalamic dysregulation of appetite (2). Understanding these mechanisms explains why lifestyle interventions, although first-line therapies, often have limited long-term effectiveness (3). This has led to the development of gut hormone-based pharmacotherapies, such as GLP-1 receptor agonists (GLP-1 RAs), that target central appetite pathways, contributing to meaningful and sustained weight loss (WL) (4).

Among these agents, semaglutide was the first to demonstrate robust efficacy in WL, primarily through appetite suppression and improved satiety, leading to expanded indications for overweight and obesity treatment. Beyond WL, semaglutide provides cardiovascular, renal, hepatic and metabolic benefits (5) that appear to be at least partially independent of WL, suggesting a drug-specific intrinsic effect (6).

These central pathophysiological mechanisms make obesity a chronic, relapsing disease, suggesting that ongoing GLP1-RA therapy is required to maintain sustained benefits (1). In real-world practice, these drugs are often less effective than in randomized clinical trials (RCTs), likely due to poor adherence, early discontinuation, or suboptimal dosing (7). These factors, along with prior GLP-1 RA exposure, sex-differences, and baseline BMI remain understudied in obesity cohort (8), particularly in real-world settings (9).

This retrospective study aimed to evaluate the early and 12-month effectiveness of once-weekly (OW) semaglutide on anthropometric and metabolic parameters in a real-world cohort of patients with overweight/obesity without type 2 diabetes (T2D), including weight loss (kg and percent) and the percentage of participants achieving weight loss targets of 5%, 10% and 15% mean at 3-month, 6-month and 12-months of follow-up, as well as non-invasive indices of hepatic steatosis and fibrosis. In addition, treatment adherence and reasons for discontinuation were assessed, along with sub-analyses of weight response by prior liraglutide exposure, sex, and baseline BMI categories.

## 2 Materials and methods

### 2.1 Study population

We conducted a retrospective, real-world, multicenter study of patients prescribed semaglutide OW between September 2024 and November 2025 at the University Obesity Unit of Santa Maria Goretti Hospital (Latina) and the Obesity Center of Policlinico Tor Vergata University Hospital (Rome), Italy. Consecutive patients aged ≥ 18 years with a BMI ≥ 30 kg/m^2^ or a BMI ≥ 27 kg/m^2^with at least one weight-related complication (such as hypertension, prediabetes, or dyslipidemia) who remained on treatment for at least 3 months of follow-up were included in the study. Exclusion criteria included T2D (10), previous bariatric surgery, pregnancy. All patients received a hypocaloric Mediterranean diet and exercise recommendations at the time of semaglutide initiation and lifestyle counseling at each follow-up visit. Patients prescribed other dietary protocols (e.g., ketogenic diet) were excluded from the analysis.

Patients initiated semaglutide OW at dose escalations of 0.25, 0.5, 1.0, or 1.7 mg and 2.4 mg throughout the follow-up period. Dose adjustments were individualized based on patient tolerability and economic financial constraints.

### 2.2 Data collection and definition

Demographic, anthropometric, and metabolic data were collected retrospectively at baseline (T0), T1 (2–4 months), T2 (5–8 months), and T3 (9–15 months). Anthropometric measurements included body weight (BW), height, body mass index [BMI: weight (kg)/height squared (m²)], waist circumference (WC), and waist to height ratio [WHtR: WC (cm)/height (m)]. Percentage WL (%WL) and the proportion of patients achieving WL ≥5%, ≥10%, and ≥15% were calculated. Metabolic parameters included assessment of glucose metabolism [fasting blood glucose (FBG) and glycated hemoglobin (HbA1c)], lipid profile [total cholesterol, low-density lipoprotein (LDL) and high-density lipoprotein (HDL) cholesterol, and triglycerides], liver function [alanine aminotransferase (AST), alanine aminotransferase (ALT), and gamma glutamyl transferase (GGT)], and renal function [creatinine and estimated glomerular filtration rate (eGFR) calculated using the MDRD formula]. Only patients who did not discontinue therapy at any follow-up were included. As in real-world clinical practice, not all patients had complete laboratory data at all time points, so analyses were performed using the available data.

### 2.3 Non-invasive liver disease assessments and visceral adipose tissue dysfunction indices

Liver steatosis was assessed using the Fatty Liver Index (FLI) and the Hepatic Steatosis Index (HSI), while the Fibrosis-4 Index score (FIB-4) and the AST to Platelet Ratio Index (APRI) were used for liver fibrosis. All were calculated using established formulas (11). Visceral adipose tissue dysfunction was estimated using the Visceral Adiposity Index (VAI), calculated using sex-specific formulas (12).

### 2.4 Assessment of pharmacotherapy discontinuation

For the safety evaluation, data on prescribed doses, reported adverse events and whether these led to treatment discontinuation were collected from medical records at each patient visit, when available.

## 3 Statistical analysis

Quantitative variables were expressed as mean ± standard deviation (SD) for normally distributed data and as median and interquartile range (IQR) for non-normally distributed variables. Categorical variables were expressed as percentages. Treatment effectiveness at follow-up was assessed using the Student’s t-test, Mann-Whitney test, or chi-squared test, as appropriate. Within-group comparisons were performed using the Wilcoxon signed-rank test for paired sample. Between-group comparisons were performed using the Mann-Whitney U test for two independent groups and the Kruskal-Wallis H test for more than two groups, with *post-hoc* pairwise comparisons adjusted for multiple testing using the Bonferroni correction. Sub-analyses were performed by patients’ previous exposure to liraglutide (naïve versus switchers), sex, and based on baseline BMI categories (25-29.9, 30-34.9, 35-39.9, and ≥ 40 kg/m²). To assess the independent effect of achieving WL ≥5% (categorical variable) on early (T1) changes in metabolic parameters, participants were stratified by %WL (≥5% vs. <5%), and a multivariable linear regression model adjusted for age and sex was conducted. An additional model included change in WC at T1 (ΔWC) as a covariate. A p-value < 0.05 was considered statistically significant. Analyses were performed using IBM SPSS Statistics software, version 21.0.

## 4 Results

### 4.1 General and clinical characteristics of the population

**Figure 1:**
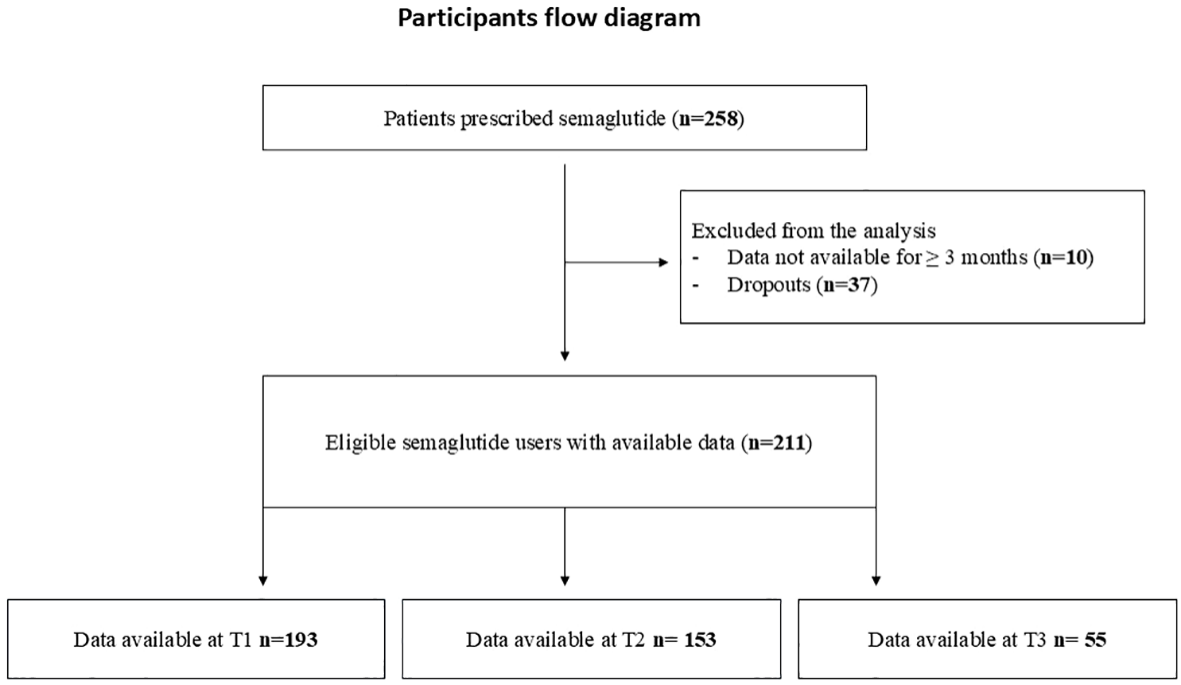
The study flow chart.

A total of 258 patients with obesity without T2D treated with OW semaglutide was initially included in the analysis. Of these, 196 (76.0%) were GLP-1 RA-naïve, while 62 (24.0%) were switching from liraglutide, with a median last maximum liraglutide dose of 1.8 (1.9-2.2) mg/day. The median age of the overall cohort was 55.0 (51.1-54.7) years, and the median BMI was 34.8 (35.2-36.6) kg/m^2^. General and clinical characteristics of the study population are summarized in **Supplementary Table 1**. Ten patients were excluded due to missing follow-up data for ≥ 3 months (**Figure 1**).

### 4.2 Change in anthropometric and metabolic parameters

**Figure 2:**
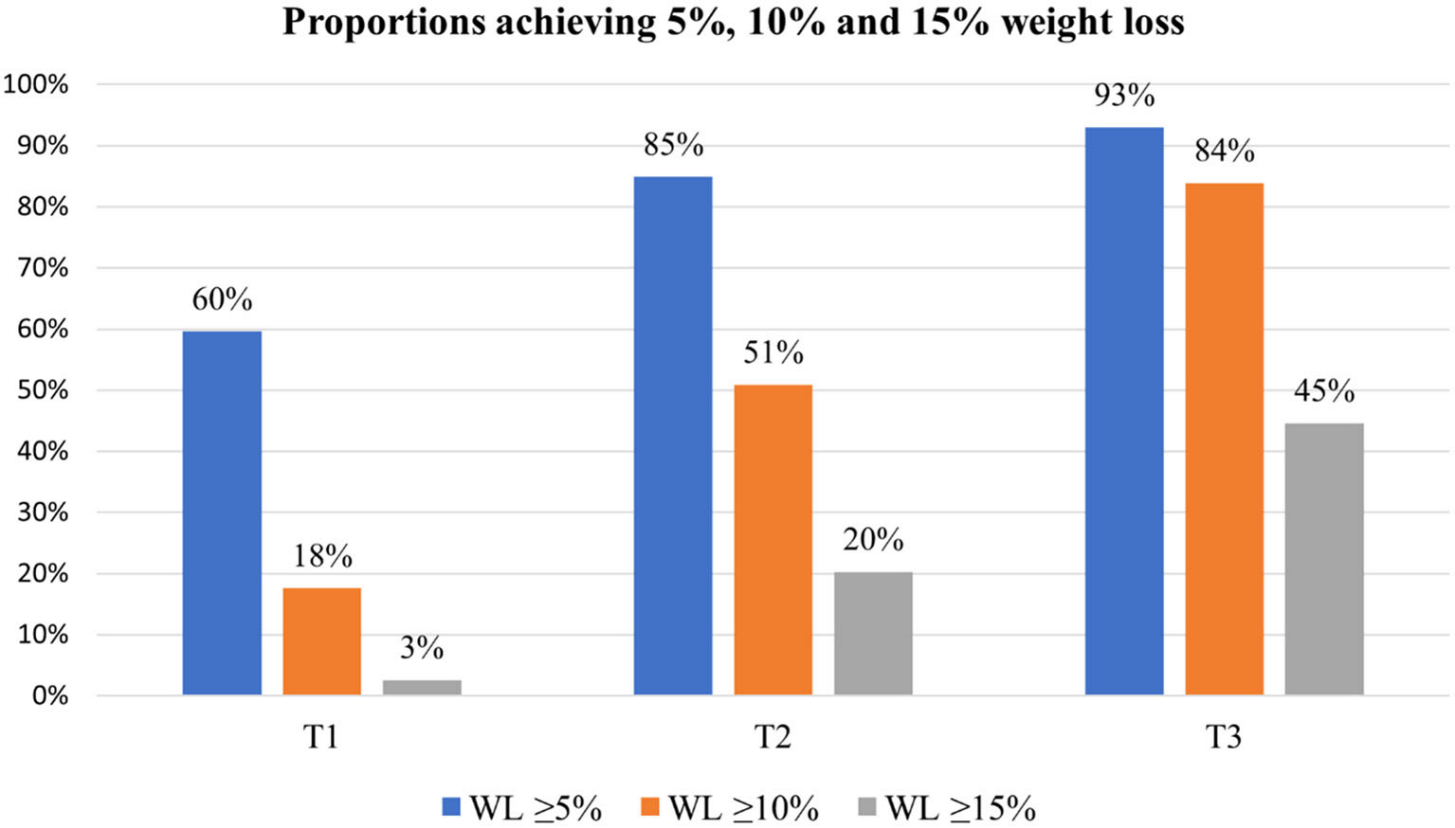
Percentage of patients achieving weight loss of ≥5%, ≥10%, and ≥15% after treatment. WL, Weight loss.

Semaglutide was associated with a significant (p < 0.001) reduction in anthropometric parameters (**Table 1**). BW data were available for 193 patients at T1, 153 at T2 and 55 at T3. At all follow-up visits, BW was significantly reduced from baseline by -5.0 kg, -9.5 kg, and -12.5 kg, corresponding to percentage weight loss of -5.3%, -9.8%, and -13.3%, respectively (**Table 1**). Specifically, WL ≥5% was achieved in 60% of patients at T1, 84.9% at T2, and 93% at T3. The percentage of patients achieving WL ≥ 10% increased from 18% at T1 to 51% and 84% at T2 and T3, respectively. Approximately 20.3% and 45% of patients also achieved WL ≥ 15% at T2 and T3, respectively (**Figure 2**). Reduction in BMI (-2.0 kg/m^2^; -3.6 kg/m^2^; -4.5 kg/m^2^) and visceral adiposity measures such as WC (-4. 0 cm; -9.0 cm; -12.5 cm) and WHtR (-0.03; -0.06; -0.08) were also observed at all follow-up time points (**Table 1**).

**Table 1:** Changes in anthropometric parameters after treatment.

Parameter	Overall	p-value
Weight(kg)		
T1-T0 N = 193	-5.0 (-6.1,-5.0)	<0.001
T2-T0 N = 153	-9.5 (-11.1,-9.2)	<0.001
T3-T0 N = 55	-12.5 (-15.7,-11.8)	<0.001
Weight(%)		
T1-T0 N = 193	-5.3 (-6.4, -5.3)	<0.001
T2-T0 N = 153	-9.8 (-14.9, -8.9)	<0.001
T3-T0 N = 55	-13.3 (-17.1, -13.5)	<0.001
BMI (kg/m^2^)		
T1-T0 N = 193	-2.0 (-2.2,-1.8)	<0.001
T2-T0 N = 153	-3.6 (-4.7,-3.3)	<0.001
T3-T0 N = 55	-4.5 (-6.6,-4.4)	<0.001
WC(cm)		
T1-T0 N = 102	-4.0 (-6.5,-4.5)	<0.001
T2-T0 N = 77	-9.0 (-11.4,-8.7)	<0.001
T3-T0 N = 34	-12.5 (-15.9,-10.9)	<0.001
WHtR		
T1-T0 N = 102	-0.03 (-0.04,-0.03)	<0.001
T2-T0 N = 77	-0.06 (-0.07,-0.05)	<0.001
T3-T0 N = 34	-0.08 (-0.10,-0.04)	<0.001

Data are expressed as median and IQR (25-75). BMI, Body Mass Index; WC, Waist Circumference; WHtR, Waist to Height Ratio; WL, Weight Loss.

Median Semaglutide dose T1-T0 = 1.0 (0.7, 0.8) mg (min. 0.25- max 1.7)

Median Semaglutide dose T2-T0 = 1.2 (1.2, 1.4) mg (min. 0.25- max 2.4)

Median Semaglutide dose T3-T0 = 1.2 (1.1, 1.4) mg (min. 0.25- max 2.4)

Changes in metabolic parameters are shown in **Table 2** with mostly significant improvements over the follow-up period in terms of glucose controls (FBG, and HbA1c), lipid profile (total, LDL and HDL cholesterol, triglycerides), and liver enzymes (AST, ALT GGT). Lipid improvements were consistent regardless of lipid-lowering therapy (**Supplementary Table 2**). Notably, NILDAs (HSI, FLI, FIB-4, APRI), and VAI index improved significantly throughout follow-up.

**Table 2:** Changes in metabolic parameters after treatment.

Parameter	Overall	P-value
FBG (mg/dL)		
T1-T0 N = 38	-9.0 (-12.8,-6.9)	**<0.001**
T2-T0 N = 36	-8.0 (-13.9,-5.1)	**<0.001**
T3-T0 N = 16	-1.5 (-9.7,2.7)	0.365
HbA1c		
T1-T0 N = 22	-0.1 (-0.25,0.05)	0.133
T2-T0 N = 28	-0.35 (-0.40,-0.15)	**<0.001**
T3-T0 N = 14	-0.40 (-0.62,-0.22)	**0.002**
Total cholesterol (mg/dL)		
T1-T0 N = 46	-12.5 (-26.1-6.4)	**<0.001**
T2-T0 N = 50	-11.0 (-30.4,-7.0)	**0.001**
T3-T0 N = 20	-28.0 (-62.0,-9.0)	**0.009**
HDL (mg/dL)		
T1-T0 N = 44	-3.0 (-3.8,3.1)	0.084
T2-T0 N = 45	-1.0 (-5.2,0.14)	0.113
T3-T0 N = 18	2.0 (-3.8,7.0)	0.522
LDL (mg/dL)		
T1-T0 N = 43	-8.0 (-23.6,-1.9)	**0.005**
T2-T0 N = 45	-9.2 (-27.6,-2.4)	**0.012**
T3-T0 N = 19	- 16.2 (-50.2,-6.0)	**0.018**
Triglycerides (mg/dL)		
T1-T0 N = 45	-5.0 (-19.9,4.6)	0.264
T2-T0 N = 47	-21.0 (-31.0,-6.7)	**0.003**
T3-T0 N = 18	-7.0 (-30.7,0.02)	0.094
Creatinine (mg/dL)		
T1-T0 N = 43	0.00 (-0.03,0.05)	0.922
T2-T0 N = 43	0.02 (-0.02,0.05)	0.067
T3-T0 N = 15	0.02 (-0.06,0.07)	0.842
MDRD (mL/min)		
T1-T0 N = 42	0.00 (-5.0,2.5)	0.910
T2-T0 N = 42	-1.43 (-5.4,0.35)	0.079
T3-T0 N = 15	-3.00 (-9.6,7.0)	0.650
AST (UI/L)		
T1-T0 N = 29	-3.0 (-10.0,-2.8)	**0.004**
T2-T0 N = 34	-2.0 (-9.7,-2.1)	**0.001**
T3-T0 N = 13	-3.0 (-16.9,1.5)	**0.045**
ALT (UI/L)		
T1-T0 N = 32	-2.0 (-15.7,6.0)	**0.045**
T2-T0 N = 34	-2.5 (-18.5,-2.3)	**0.029**
T3-T0 N = 13	-3.0 (-26.9,2.7)	0.074
GGT (UI/L)		
T1-T0 N = 25	-2.0 (-4.1,2.9)	0.079
T2-T0 N = 24	-2.5 (-4.9,2.4)	0.121
T3-T0 N = 11	-8.0 (-10.9,-4.0)	**0.009**
HSI		
T1-T0 N = 31	-2.3 (-10.3,-2.5)	**<0.001**
T2-T0 N = 32	-3.0 (-5.0,-2.5)	**<0.001**
T3-T0 N = 13	-4.7 (-13.7,-2.3)	**<0.001**
FLI		
T1-T0 N = 17	-7.4 (-23.5,-2.4)	**<0.001**
T2-T0 N = 11	-23.4 (-34.3,-13.2)	**0.001**
T3-T0 N = 7	-45.4 (-66.6,-10.8)	**0.012**
FIB-4		
T1-T0 N = 24	-0.18 (-0.32,-0.05)	**0.005**
T2-T0 N = 23	-0.10 (-0.27,0.04)	**0.045**
T3-T0 N = 9	-0.11 (-0.47,0.12)	**0.047**
APRI		
T1-T0 N = 34	-0.05 (-0.13,-0.02)	**0.002**
T2-T0 N = 35	-0.02 (-0.13,-0.01)	**0.025**
T3-T0 N = 12	-0.03 (-0.13,-0.06)	**0.041**
VAI		
T1-T0 N = 40	-0.36 (-1.0,-0.32)	**<0.001**
T2-T0 N = 42	-0.13 (-0.70,-0.14)	**0.006**
T3-T0 N = 18	-0.66 (-1.0,-0.30)	**0.008**

Data are expressed as median and IQR (25-75). HbA1c, glycated hemoglobin; FBG, fasting bood glucose; HDL, high-density lipoprotein cholesterol; LDL, low-density lipoprotein cholesterol; MDRD, Modification of Diet in Renal Disease equation; AST, aspartate aminotransferase; ALT, alanine aminotransferase; GGT, gamma-glutamyl transferase; HSI, hepatic steatosis index; FLI, fatty liver index; FIB-4, fibrosis-4 index; APRI, AST-to-platelet ratio index; VAI, visceral adiposity index.

Statistically significant p-values (p<0.05) are shown in bold in the table.

Although early metabolic changes at T1 tended to be greater in participants who achieved WL≥5%, the differences were not statistically significant (**Supplementary Table 3**). Consistently, multivariable linear regression (**Supplementary Table 4**) showed that early metabolic changes were independent of WL ≥5% after age-and-sex adjustment Results remained unchanged after including ΔWC, which correlated significantly with HSI (β=-0.527, 95% CI -1.5 to -0.08, p=0.032) and showed a trend for FIB-4 (β=-0.481, 95% CI -0.419 to 0.002, p=0.052). After further adjustment for hypolipidemic therapy, %WL ≥5% was only independently associated with changes in total (β=-0.393, 95% CI -49.1 to 0.552, p=0.055) and LDL (β=-0.442, 95% CI -49.0 to -2.3, p=0.033) cholesterol, supporting a WL-dependent improvement in lipid profile regardless of pharmacological therapy (**Supplementary Table 5**).

### 4.3 Analysis by prior GLP-1 RAs exposure (naïve vs switchers)

Although baseline anthropometric characteristics were similar (**Supplementary Table 6**), naïve patients showed greater reductions in BW (-6.0 vs -2.7; -10.5 vs -6.5, p < 0.001 kg), %WL (-6.0 vs -2.7; -11.0 vs -7.5, p < 0.001%), BMI (-2.2 vs -0.9; -3.9 vs -2.6, p < 0.001 kg/m^2^), WC (-4.5 vs -3.0, p = 0.043 cm; -10.0 vs -8.5, p = 0.047 cm), and WHtR (-0.03 vs -0.02, p = 0.036; -0.06 vs -0.05, p = 0.048) at T1 and T2 compared with patients switching from liraglutide. Comparable effectiveness was observed for all parameters at T3 (**Table 3**).

**Table 3:** Changes in anthropometric parameters after treatment according to prior GLP-1 RAs exposure (naïve vs switchers).

Parameter	Naïve	Switchers	P-value
Weight(kg)			
T1-T0	-6.0 (-6.9, -5.6) N=147	-2.7 (-3.9, -2.3) N=46	**<0.001**
T2-T0	-10.5 (-12.2,-10.0) N=119	-6.5 (-8.1, -5.2) N= 34	**<0.001**
T3-T0	-12.3 (-17.0,-12.3) N=38	-12.6 (-15.1, -8.0) N=17	0.372
Weight(%)			
T1-T0	-6.0 (-7.2, -5.8)	-2.7 (-4.6, -2.7)	**<0.001**
T2-T0	-11.0 (-17.0, -9.3)	-7.5 (-9.1, -5.9)	**<0.001**
T3-T0	-13.6 (-18.0, -13.6)	-13.3 (-17.6, -11.0)	0.402
BMI (kg/m2)			
T1-T0	-2.2 (-2.5, -2.1) N=147	-0.9 (-1.4, -0.9) N=46	**<0.001**
T2-T0	-3.9 (-5.3,-3.6) N=119	-2.6 (-3.1, -2.0) N= 34	**<0.001**
T3-T0	-4.5 (-6.3,-4.6) N=38	-4.5 (-8.7, -2.5) N=17	0.477
WC (cm)			
T1-T0	-4.5 (-7.6,-4.9) N= 91	-3.0 (-5.0, -2.8) N= 39	**0.043**
T2-T0	-10.0 (-12.9,-9.2) N=51	-8.5 (-9.9, -6.3) N=26	**0.047**
T3-T0	-15.0 (-18.0, -11.8) N=23	-12.0 (-14.5, -5.8) N=11	0.123
WHtR			
T1-T0	-0.03 (-0.05,-0.03) N= 67	-0.02 (-0.03, -0.02) N= 35	**0.036**
T2-T0	-0.06 (-0.08,-0.06) N=51	-0.05 (-0.06, -0.04) N=26	**0.048**
T3-T0	-0.09 (-0.10,-0.06) N=23	-0.07 (-0.10, 0.04) N=11	0.133

Data are expressed as median and IQR (25-75). BMI, Body Mass Index; WC, Waist Circumference; WHtR, Waist to Height Ratio; WL, Weight Loss.

Statistically significant p-values (p<0.05) are shown in bold in the table.

A higher proportion of naïve patients achieved WL ≥ 5% at T1-T2 (70 vs. 28.2%, p < 0.001; 89.1 vs. 70.6%, p = 0.021) and WL ≥ 10% at T2 (57.9 vs. 26.5%, p = 0.002), with a trend for WL≥ 10% at T1 and WL≥ 15 at T2, but non-significant. No differences were observed at T3 (**Supplementary Figure 1**), indicating that weight loss outcomes at 12 months with semaglutide were independent of prior exposure to liraglutide.

### 4.4 Analysis according to sex-differences

Women were overrepresented compared to men (77.5% vs. 22.5%) and had lower dropout rates (11.0% vs. 25.8%, p = 0.005). Although men had significantly (p < 0.001) higher baseline BW [112.5 (109.1-119.5) vs. 90.7 (91.6-96.0) kg] and WC [122.5 (115.1-126.1) vs. 106.0 (105.0-109.0) cm] than women (**Supplementary Table 1**), no significant sex-differences were observed for changes in all anthropometric parameters over time (**Supplementary Table 7**) or in the proportions of patients achieving WL ≥ 5%, ≥ 10%, and ≥ 15% at any of the three time points (**Supplementary Figure 2**).

### 4.5 Analysis according to baseline BMI categories

No significant differences were observed across baseline BMI categories in anthropometric outcomes (**Supplementary Table. 8**) or in the proportions of patients achieving ≥ 5%, ≥ 10%, and ≥ 15% WL at any of the three time points (**Supplementary Figure 3**). All comparisons remained non-significant after Bonferroni adjustment.

### 4.6 Discontinuation, adverse events and reasons for discontinuation

Among 248 patients, 37 (14.9%) were classified as dropouts because they did not start semaglutide therapy or were lost to follow-up immediately after prescription. Of the 211 patients who started treatment, 103 (48%) patients discontinued treatment within the first year of follow-up. Adverse events led to discontinuation in only 22 patients (10.4%), while other reasons for discontinuation included insufficient weight loss (10.9%), loss to follow-up (7.6%), cost (5.7%), stopping treatment after reaching desired body weight (5.2%), switching to another anti-obesity treatment (4.7%), and personal reasons of the patient (2.8%). The remaining patients discontinued the treatment for unspecified reasons (1.4%). Among them, ninety patients (42.6%) reported a total of 131 mild to moderate adverse events that were mostly gastrointestinal (90%), followed by myalgia, arthralgia and asthenia (**Table 4**).

**Table 4:** Adverse events associated with semaglutide treatment.

Adverse event	N = 131 (%)
Gastrointestinal side effects	118 (90.0%)
Nausea and vomiting	52 (40.0%)
Gastroesophageal reflux disease	16 (12.2%)
Constipation	24 (18.3%)
Diarrhea	16 (12.2%)
Abdominal discomfort and distention	10 (7.6%)
Myalgia and arthralgia	3 (2.3%)
Asthenia	3 (2.3%)
Others	7 (5.3%)

Data are expressed as numbers and percentages [n (%)].

Among others (5.3%), only one patient reported elevated amylase and lipase without signs of pancreatitis. One patient reported periorbital edema without other allergic symptoms. Only one patient experienced mood changes, but this individual had a pre-existing psychiatric disorder. One patient reported increased hair loss, while another patient reported headache with scotoma not clearly related to drug administration. All reported adverse events were non-severe, transient or reversible after temporary discontinuation of therapy. No serious adverse events occurred, further supporting the favorable safety and tolerability profile of semaglutide.

## 5 Discussion

This retrospective, real-world study evaluated the effectiveness, tolerability, and adherence of semaglutide after its approval in Italy in July 2024. Our baseline cohort characteristics were largely comparable to those reported in RCTs of obesity medications, with the exception of a higher median age and slightly lower baseline BMI compared to both RCTs (13, 14) and other real-world studies (15–20).

### 5.1 Effectiveness

This retrospective, real-world study shows that treatment with semaglutide was associated with substantial and sustained weight loss over 12 months of follow-up in adults with obesity without T2D, with %WL values of 5.3%, 9.8% and 13.3% at approximately 3, 6 and 12 months, respectively. Notably, in addition to the consistently high proportion of patients achieving at least 5% weight loss, weight loss of more than 10% was also observed early and increased throughout follow-up, suggesting a durable treatment effect.

The observed weight loss, both in absolute terms and as a percentage of baseline body weight, is comparable to that reported in previous real-world studies (15, 16, 18, 20), while increasing the evidence for the effectiveness and persistence of treatment in individuals with obesity without T2D in routine clinical care. Indeed, with the exception of Tzoulis et al. (16), these real-world studies also included patients with T2D, which limits the applicability of their results to people with obesity without T2D. On the other hand, one real-world study reported slightly greater weight loss (approximately 15.4% at 12 months) (19); however, it included patients who successfully escalated to and maintained the recommended 2.4 mg maintenance dose and had private health insurance, limiting the generalizability of the results to broader populations.

Notably, these significant WLs were achieved in our cohort at approximately half the approved maintenance dose of 2.4 mg. Previous real-world evidence showed that between 33% and 48% of patients prescribed semaglutide remained on the 1.0 mg dose (21). In addition, another real-world study reported that only 24% of patients received doses ≥2 mg by the sixth prescription, with mean weight loss of 5.7% at three months, 9.8% at six months, and 15.6% at one year (20). On the other hand, in contrast to the obesity semaglutide RCTs (22), including STEP1 (13) and STEP5 (14), which used structured dose escalation, intensive lifestyle intervention, and enrolled only patients who had discontinued anti-obesity medications before study entry, dose escalation was less frequent in our cohort, and not all patients were GLP-1 RA-naïve. This better reflects real-world setting (17). Given that STEP1 patients achieved weight loss of approximately 6%, 10% and 14% at mean 3, 6 and 12 months, our results support the effectiveness of semaglutide beyond the controlled conditions of RCTs. In addition, our results extend previous real-world evidence of clinically meaningful weight loss even at lower than maximum maintenance doses in the obesity cohort.

Secondary endpoint results from RCTs (22), as well as findings from a real-world study (19), have shown improvements in cardiometabolic risk factors after long-term treatment with semaglutide 2.4 mg. A novel finding of our study is that these improvements, which extend to non-invasive indices of liver steatosis, fibrosis and visceral adiposity, occurred early after treatment initiation and at lower doses. Furthermore, although the limited 12-month data available warrant cautious interpretation, these improvements were sustained over the long term, supporting the metabolic benefits of semaglutide treatment. Notably, the lack of an independent association between achieving ≥5% weight loss and the early metabolic changes suggests that the relationship between weight loss and metabolic improvement is more complex than simply achieving a predefined weight loss threshold. In contrast, the independent association between waist circumference and non-invasive liver steatosis and fibrosis scores suggests that central adiposity may be particularly relevant to liver-related improvements. Given the limited sample size in some adjusted analyses, particularly those involving liver-related biomarkers, prospective studies with larger cohorts, longer follow-up, and standardized collection of metabolic biomarkers are warranted to validate and extend our findings. However, the observed early metabolic improvements that are not entirely attributable to WL may have potential clinical implications that support a broader approach to obesity management beyond predefined weight loss thresholds, including sustained weight loss and management of obesity-related comorbidities (5, 8). Therefore, future studies should aim to identify distinct obesity phenotypes and evaluate the use of semaglutide using primary endpoints other than weight loss, such as progression or regression of liver disease (16).

### 5.2 Liraglutide switching

To date, the limited evidence on clinical outcomes after switching within the GLP-1 RA class has focused on patients with obesity and T2D (18, 23). Our results extend these findings by demonstrating additional WL after switching from liraglutide to semaglutide in patients with obesity without T2D. We also observed that prior exposure to liraglutide influenced the timing of WL: naïve patients typically achieved their maximum WL within approximately 6 months, whereas switchers showed an attenuated early response, requiring up to 12 months to achieve comparable results, as observed in T2D populations (24, 25).

Given similar baseline characteristics, several factors could account for this lower early response in switchers, such as the WL plateau (“set point”) often seen after obesity treatments (26, 27) or metabolic, biological, or receptor level adaptations associated with prior exposure to the same pharmacological class (28). The gradual dose titration recommended for this pharmacotherapy may also have resulted in underdosing after switching, thereby limiting early WL (29). In fact, although switchers received slightly higher doses at T1, these remained below the recommended maintenance dose for obesity with both groups exhibiting sub-target dosing after 12 months. This lower dose may be particularly relevant in previously treated patients, as supported by exposure-response modeling suggesting that higher initial doses of semaglutide may prevent early worsening of treatment outcomes (30). Although general expert recommendations for switching between GLP-1 RAs exist in diabetes management (31), there are currently no specific guidelines for obesity. Since dose adjustments are often individualized based on tolerability rather than prior weight history, these findings highlight the need for studies to define optimal dosing strategies in future guidelines.

### 5.3 Sex differences

It is widely recognized the existence of a sexual dimorphism in obesity prevalence and treatment (32). The term sex refers to biological characteristics, such as hormones, chromosomes, and reproductive anatomy, while gender refers to socially constructed roles, behaviors, and identities that vary across cultures and time (33). The overrepresentation of women in our study reflects those reported in obesity RCTs (34), and real-world data confirming that women seek and use semaglutide for WL more frequently than men (35, 36). Higher dropout rates among men may be due to their lower awareness of obesity as a chronic disease, which reduces the likelihood of perceiving excess weight as a condition requiring medical care (37). Although greater WL has been reported in women receiving anti-obesity pharmacotherapy, including semaglutide (8, 38), we found no sex-differences in anthropometric outcomes. Similarly, although one real-world study found a higher distribution of non-responders in men than in women, this result did not reach statistical significance, nor was there a significant difference in three-month weight change between the sexes (16). Furthermore, the SELECT trial confirms clinically meaningful WL in both sexes (27). A recent large-scale analysis found no substantial qualitative sex-differences in overall treatment response, suggesting that mixed-age cohorts may obscure sex-differences that are predominantly present in premenopausal women (39). Mechanisms underlying sex-related differences, including sex hormones, fat distribution, gastric emptying, and central food regulation (32) are influenced by menopause-related physiological changes (40). Evidence suggests that sex hormones may modulate GLP-1 signaling and treatment efficacy in postmenopausal women (39, 40), with studies reporting greater responses in women mainly including premenopausal participants (41). Conversely, in middle-aged patients with obesity without T2D, liraglutide was associated with greater WL and cardiometabolic improvements in men (42). These findings highlight the value of age-stratified studies for identifying sex-differences in response (39), while noting that men seeking obesity treatment in real-world settings may be particularly motivated to adhere to therapy (37).

### 5.4 Baseline BMI categories

Our findings align with both real-world (16, 41, 43) and RCTs (27, 44) data, indicating that semaglutide effectiveness is largely independent of baseline BMI. Conversely, baseline BMI appears to be particularly relevant in individuals below the obesity threshold, as suggested by the SELECT trial, which reported a more attenuated weight loss response in this subgroup compared with participants with obesity, while weight loss was broadly similar across obesity classes (I–III) (27). It can be hypothesized that in individuals with lower BMI (e.g., ≤ 30 kg/m^2^), reduction of dysfunctional or ectopic fat may be more relevant to metabolic health than absolute WL (27). Moreover, comparable improvements across BMI categories have been reported for inflammatory markers and cardiac function (45, 46). As a result, current European prescribing criteria based primarily on BMI thresholds may exclude patient subgroups that could potentially benefit from therapy (44). Although BMI is used to determine eligibility for anti-obesity pharmacotherapy, it remains a poor predictor of both treatment response and obesity-related health risks (47). Therefore, these findings support a shift from a BMI-centered to a risk-based, individualized strategy for anti-obesity pharmacotherapy (48).

### 5.5 Persistence and safety

As treatment patterns in obesity continue to evolve rapidly, continuously updated population-based drug utilization data are needed, as currently available real-world evidence remains limited, particularly with respect to treatment persistence, reasons for discontinuation, and adverse events in the non-T2D obesity cohort (17). Although the first-year discontinuation rates observed in this study were higher than those reported in RCTs (22), this trend is consistent with recent real-world data reporting high variability in discontinuation rates, ranging from 20 to 60% within the first year of treatment (7, 17, 49), especially in obesity cohort without T2D. This heterogeneity in the everyday clinical care has been attributed to several factors, including physician-patient barriers, side effects, and the lack of reimbursement for these therapies (7, 17). Notably, consistent with findings from RCTs and real-world studies, no serious adverse events were reported in our cohort. Most adverse events were mild to moderate in severity, with nausea and vomiting being the most common. Although only a small proportion of patients discontinued treatment due to adverse events, consistent with the established safety profile of the treatment reported in RCTs (22), these rates were slightly higher compared to other real-world studies (15–17). This finding may be explained by the limited follow-up typical of community-based care, which may delay timely management of intolerable side effects, and by the older age of our cohort, which may increase susceptibility to gastrointestinal adverse events (50). On the other hand, the SELECT studies reported higher discontinuation rates with semaglutide in lower BMI classes (27). Therefore, the slightly lower BMI of our cohort may have partially contributed to this different rate. Regarding financial reasons, interestingly, few patients discontinued treatment due to cost, suggesting that the recent legal recognition of obesity as a disease in Italy (51) may have facilitated treatment initiation, even in real-world settings with limited financial resources, reflecting the real-world context of obesity (37). On the other hand, some patients were lost to follow-up immediately after prescription, while others discontinued treatment after achieving satisfactory weight loss. Consistent with this, a real-world study reported that 50% of patients discontinued GLP-1 RA therapy within 12 months, despite insurance coverage, suggesting that supply constraints, tolerability issues, and, in particular, patients’ desire to discontinue treatment after achieving weight loss may contribute to these high discontinuation rates (20). Therefore, our findings suggest that in real-world clinical practice, in addition to differences in baseline patient characteristics, limited awareness of obesity as a chronic, relapsing disease, together with financial constraints, may contribute to suboptimal long-term treatment adherence in obesity.

This study has several limitations. The retrospective design resulted in missing data for some outcomes. Medical record data were not always complete for all patients and at all time points. As an observational study, residual confounding due to unmeasured variables cannot be excluded. Heterogeneity in semaglutide dosing and treatment exposure may also have influenced the observed results. Treatment discontinuation and loss to follow-up reduced the number of patients available for long-term evaluation, which may have limited the robustness of these analyses. In addition, exclusion of patients who discontinued treatment or were lost to follow-up before the first scheduled assessment may have introduced a survivorship bias. Finally, some adjusted analyses, particularly those involving liver-related biomarkers, were limited by small sample sizes, which may reduce statistical robustness.

Strengths of this study include the comprehensive collection of clinical, anthropometric, dosing, and metabolic data, allowing a detailed assessment of the early and 12-month effectiveness of semaglutide in a real-world obesity cohort without T2D, an area where data remain limited. The real-world design and subgroup analyses based on prior liraglutide use, sex and baseline BMI categories provide insights that complement those from RCTs by better reflecting routine obesity management, particularly with respect to treatment persistence, dose escalation, adverse events, reasons for discontinuation and switching from prior GLP-1 receptor agonists. These findings are particularly relevant given the current lack of clinical guidance on the long-term management of treatment discontinuation and switching in routine practice.

## 6 Conclusions

In conclusion, these results confirm the effectiveness of semaglutide in achieving significant weight reduction and improving visceral adiposity and cardiometabolic risk in a real-world obesity setting, with a favorable safety and tolerability profile. This clinically meaningful weight loss was achieved despite relatively modest maintenance doses of semaglutide supporting the effectiveness of treatment even when the maximum maintenance dose is not reached. Early metabolic improvements, independent of the 5% WL target, suggest that factors beyond weight loss contribute to effectiveness and support evaluation of treatment continuation beyond weight loss alone. The lower early response in liraglutide switchers compared to naive patients, but similar long-term effectiveness, suggests potential for dose optimization after switching to avoid suboptimal initial effects. The lack of differences by sex or baseline BMI highlights the need for studies stratified by age and metabolic risk, rather than BMI alone, to enable individualized treatment and reduce weight-related complications. On the other hand, the discontinuation rates seen in this study, which are consistent with the real-world setting, highlights the need for a patient-centered approach to obesity management that combines multidisciplinary teams and more frequent support to improve adherence and reduce the risk of discontinuation.

## Data Availability

The raw data supporting the conclusions of this article will be made available by the authors, without undue reservation.
