# Supplementary material for: Real-world semaglutide in obesity cohort: effectiveness, safety and persistence across sex, BMI, and prior GLP-1RA treatment
Source: Front Endocrinol (Lausanne). 2026 Jul 28;17:1819337. doi: 10.3389/fendo.2026.1819337 (PMC13457613; doi:10.3389/fendo.2026.1819337)
Supplement: Supplementary file 1 [file SupplementaryFile1.docx]

Supplementary Material

1. Supplementary Figures and Tables

## 1.1 Supplementary Tables

**Table S1.** General and clinical baseline characteristics of all patients and by sex.

|  | Overall n=258 | Women | Men | P-value |
| --- | --- | --- | --- | --- |
| Sex, n (%) |  | 200 (77.5%) | 58 (22.5%) | **< 0.001** |
| Age (years) | 55.0 (51.2-54.7) | 55.0 (50.7-54.8) | 55.0 (49.7-57.1) | 0.996 |
| Weight (kg) | 94.1 (96.1-100.7) | 90.7 (91.6-96.0) | 112.5 (109.1-119.5) | **< 0.001** |
| BMI (kg/m^2^) | 34.8 (35.2-36.6) | 34.1 (34.8-36.4) | 36.1 (35.5-38.6) | 0.106 |
| WC (cm) | 108.0 (107.3-11.4) | 106.0 (105.0-109.0) | 122.5 (115.1-126.1) | **< 0.001** |
| WHtR | 0.66 (0.60-0.66) | 0.66 (0.60-0.66) | 0.67 (0.53-0.71) | 0.425 |
| FBG (mg/dL) | 92.0 (90.4-94.0) | 92.0 (90.5-94.3) | 91.0 (84.9-97.3) | 0.453 |
| HbA1c (%) | 5.6 (5.2-6.7) | 5.6 (5.5-5.7) | 5.6 (5.3-6.0) | 0.250 |
| Total cholesterol (mg/dL) | 186.0 (181.5-192.2) | 191.0 (186.3-197.9) | 171.0 (154.5-178.1) | **< 0.001** |
| LDL (mg/dL) | 109.4 (105.7-115.6) | 113.0 (108.3-119.3) | 95.0 (88.1-109.8) | **0.019** |
| HDL (mg/dL) | 52.0 (52.5-56.4) | 54.0 (54.7-58.9) | 46.0 (41.9-48.5) | **< 0.001** |
| Triglycerides (mg/dL) | 106.0 (105.7-121.2) | 106.0 (102.1-117.9) | 107.0 (104.2-149.8) | 0.396 |
| Creatinine (mg/dL) | 0.78 (0.79-0.86) | 0.73 (0.75-0.82) | 0.93 (0.92-1.0) | **< 0.001** |
| MDRD (mL/min) | 82.0 (78.9-84.5) | 82.0 (78.1-84.4) | 82.2 (76.7-90.4) | 0.696 |
| AST (UI/L) | 22.0 (22.2-25.6) | 21.0 (21.3-25.0) | 24.5 (23.1-32.0) | **0.014** |
| ALT (UI/L) | 21.0 (23.2-28.7) | 22.0 (21.2-27.0) | 27.0 (27.1-43.1) | **< 0.001** |
| GGT (UI/L) | 22.0 (23.5-29.0) | 20.0 (21.5-26.6) | 28.0 (26.9-45.0) | **0.001** |
| HSI | 42.7 (42.6-44.7) | 42.7 (42.7-45.2) | 42.6 (40.1-44.0) | 0.135 |
| FLI | 80.5 (66.0-77.1) | 81.0 (66.4-77.9) | 78.2 (49.6-87.5) | 0.660 |
| FIB-4 | 0.95 (0.92-1.11) | 0.94 (0.9-1.1) | 1.0 (0.8-1.3) | 0.567 |
| APRI | 0.20 (0.19-0.23) | 0.19 (0.18-0.23) | 0.26 (0.20-0.30) | **0.009** |
| VAI | 1.05 (1.02-1.38) | 1.13 (1.0-1.4) | 0.72 (0.56-1.4) | 0.177 |
| Hyperthension theraphy, n (%) | 97 (37.6) | 81/200 (40.5%) | 16/58 (27.6%) | 0.384 |
| Lipid-lowering theraphy, n (%) | 116 (44.9) | 92/200 (46.0%) | 24/58 (41.4%) | 0.727 |
| GLP1-RA naïve, n (%) | 196 (76.0%) | 48/200 (24.0%) | 14/58 (24.1%) | 0.983 |

Parametric data are expressed as mean ± SD, nonparametric data are expressed as median and IQR (25-75), and categorical variables are expressed as number and percentage [n (%)]. BMI, Body Mass Index; WC, Waist Circumference; WHtR, Waist to Height Ratio; FBG, fasting blood glucose; HbA1c, glycated hemoglobin; LDL, low-density lipoprotein cholesterol; HDL, high-density lipoprotein cholesterol; MDRD, estimated glomerular filtration rate (eGFR) using the MDRD formula; AST, Aspartate aminotransferase; ALT, Alanine aminotransferase; GGT, Gamma-glutamyl transferase; HSI, Hepatic steatosis index; FLI, Fatty liver index; FIB-4, Fibrosis-4 index; APRI, Aspartate aminotransferase to platelet ratio index; VAI, Visceral Adiposity Index; GLP-1 RA, GLP-1 receptor agonist.

**Table S2.** Changes in lipid profile according to lipid-lowering therapy.

|  | Lipid-lowering therapy | No lipid-lowering therapy |  |
| --- | --- | --- | --- |
| Total cholesterol (mg/dL) |  |  |  |
| T1-T0 | -12.5 (-29.4-10.3) | -19.0 (-36.6, -3.5) | 0.964 |
| T2-T0 | -5.0 (-25.4, 3.3) | -20.5 (-54.3,-11.4) | **0.044** |
| T3-T0 | -29.0 (-73.2,-4.4) | -20.0 (-84.5,-25.9) | 0.684 |
| HDL (mg/dL) |  |  |  |
| T1-T0 | -3.0 (-5.9, 0.01) | -2.0 (-3.6, 11.7) | 0.116 |
| T2-T0 | -4.0 (-7.7,0.5) | 2.0 (-4.7, 3.6) | 0.215 |
| T3-T0 | -1.0 (-8.9,4.5) | 9.0 (2.5, 14.8) | **0.022** |
| LDL (mg/dL) |  |  |  |
| T1-T0 | -13.0 (-27.8, -6.8) | -7.0 (-34.2, 3.8) | 0.691 |
| T2-T0 | -4.0 (-26.1,8.0) | -12.9 (-47.8,-5.6) | 0.199 |
| T3-T0 | - 17.1 (-56.7,-4.3) | -14.0 (-76.0,28.2) | 0.550 |

Data are expressed as median and IQR (25-75).; HDL, high-density lipoprotein cholesterol; LDL, low-density lipoprotein cholesterol.

**Table S3.** Changes in metabolic parameters according to %WL≥5% achievement at T1.

|  | %WL≥5% | %W<L5% | p-value |
| --- | --- | --- | --- |
| Total cholesterol (mg/dL) | -18.0 (-33.8,-12.1) | -5.0 (-26.1,15.8) | 0.173 |
| LDL cholesterol (mg/dL) | -13.0 (-30.4,-10.2) | -1.5 (-26.1,28.5) | 0.076 |
| AST (UI/L) | -5.0 (-13.2,-3.03) | -2.0 (-13.1,24.7) | 0.205 |
| ALT (UI/L) | -3.0 (-22.0,-2.8) | -1.0 (-18.9,40.5) | 0.201 |
| GGT (UI/L) | -3.0 (-7.2,4.6) | 1.0 (-2.4,3.5) | 0.055 |
| HSI | -2.7 (-9.1,-1.6) | -2.0 (-4.1,0.3) | 0.317 |
| FLI | -9.8 (-15.1,-5.7) | -5.9 (-55.5,16.5) | 0.368 |
| FIB-4 | -0.23 (-0.33, -0.05) | -0.05 (-0.3,0.2) | 0.265 |
| APRI | -0.097 (-0.16,-0.03) | -0.038 (-0.15,0.1) | 0.483 |
| VAI | -0.63 (-1.2, -0.4) | -0.20 (-0.83,0.14) | 0.221 |

Data are expressed as median and IQR (25-75). LDL, low-density lipoprotein cholesterol; AST, aspartate aminotransferase; ALT, alanine aminotransferase; GGT, gamma-glutamyl transferase; HSI, hepatic steatosis index; FLI, fatty liver index; FIB-4, fibrosis-4 index; APRI, AST-to-platelet ratio index; VAI, visceral adiposity index.

**Table S4.** Age- and sex-adjusted linear regression coefficients of associations between early changes in metabolic parameters (T1) and weight loss ≥5% achievement (categorical variable).

| Dependent variable | Independent variable | β (95% CI) | p value | Notes |
| --- | --- | --- | --- | --- |
| AST (UI/L) | **%WL ≥5%** | −0.360 (−27.2 to 1.2) | 0.070 | — |
| ALT (UI/L) | **%WL ≥5%** | −0.326 (−44.7 to 2.7) | 0.080 | — |
| GGT (UI/L) | **%WL ≥5%** | −0.067 (−8.0 to −5.6) | 0.722 | — |
| Total cholesterol (mg/dL) | **%WL ≥5%** | −0.260 (−35.0 to 4.3) | 0.123 | Adjusted for hypolipidemic therapy |
| LDL cholesterol (mg/dL) | **%WL ≥5%** | −0.270 (−41.4 to 5.3) | 0.125 | Adjusted for hypolipidemic therapy |
| HSI | **%WL ≥5%** | −0.284 (−10.1 to 1.6) | 0.154 | — |
| FLI | **%WL ≥5%** | −0.296 (−13.8 to 38.6) | 0.323 | — |
| FIB-4 | **%WL ≥5%** | −0.350 (−0.436 to 0.048) | 0.110 | — |
| APRI | **%WL ≥5%** | −0.232 (−0.197 to 0.042) | 0.194 | — |
| VAI | **%WL ≥5%** | −0.187 (-1.15 to 0.320) | 0.260 | — |

β, beta coefficients; CI, confidence interval; %WL, percentage of weight loss; ΔWC, change in waist circumference; AST, aspartate aminotransferase; ALT, alanine aminotransferase; GGT, gamma-glutamyl transferase; LDL, low-density lipoprotein cholesterol; HSI, hepatic steatosis index; FLI, fatty liver index; FIB-4, fibrosis-4 index; APRI, AST-to-platelet ratio index; VAI, visceral adiposity index.

**Table S5.** Linear regression coefficients of associations between changes in early metabolic parameters (T1) and weight loss ≥5% achievement (categorical variable) adjusted for age, sex, and waist circumference changes at T1 (ΔWC).

| Dependent variable | Independent variable | Model | β (95% CI) | p value | Notes |
| --- | --- | --- | --- | --- | --- |
| AST (UI/L) | **%WL ≥5%** | 2 | −0.408 (−17.44 to 1.641 | 0.098 | — |
| AST (UI/L) | **ΔWC** | 2 | −0.061 (−1.425 to 1.802 | 0.806 | — |
| ALT (UI/L) | **%WL ≥5%** | 2 | −0.234 (−0.95 to 0.13) | 0.236 | — |
| ALT (UI/L) | **ΔWC** | 2 | −0.383 (−0.193 to 4.766) | 0.068 | — |
| GGT (UI/L) | **%WL ≥5%** | 2 | −0.321 (−7.248 to 1.677) | 0.200 | — |
| GGT (UI/L) | **ΔWC** | 2 | −0.056 (−0.798 to 0.644) | 0.821 | — |
| Total cholesterol (mg/dL) | **%WL ≥5%** | 2 | -0.393 (-49.1 to 0.552) | **0.055** | Adjusted for hypolipidemic therapy |
| Total cholesterol (mg/dL) | **ΔWC** | 2 | 0.104 (-3.446 to 5.361) | 0.657 | Adjusted for hypolipidemic therapy |
| LDL cholesterol (mg/dL) | **%WL ≥5%** | 2 | -0.442 (-49.04 to -2.33) | **0.033** | Adjusted for hypolipidemic therapy |
| LDL cholesterol (mg/dL) | **ΔWC** | 2 | 0.175 (-2.42 to 5.45) | 0.428 | Adjusted for hypolipidemic therapy |
| HSI | **%WL ≥5%** | 2 | -0.326 (-7.1 to 1.3) | 0.153 | — |
| HSI | **ΔWC** | 2 | -0.527 (-1.470 to -0.08) | **0.032** | — |
| FLI | **%WL ≥5%** | 2 | -0.312 (-11.2 to 3.7) | 0.288 | — |
| FLI | **ΔWC** | 2 | 0.424 (-0.6 to 2.1) | 0.231 | — |
| FIB-4 | **%WL ≥5%** | 2 | -0.481 (-0.419 to 0.002) | **0.052** | — |
| FIB-4 | **ΔWC** | 2 | -0.024 (-0.42 to 0.04) | 0.930 | — |
| APRI | **%WL ≥5%** | 2 | -0.172 (-0.165 to 0.073) | 0.431 | — |
| APRI | **ΔWC** | 2 | -0.149 (-0.015 to 0.028) | 0.526 | — |
| VAI | **%WL ≥5%** | 2 | 0.040 (−1.222 to 1.356) | 0.915 | Adjusted for interaction %WL ≥5%*sex |
| VAI | **ΔWC** | 2 | −0.049 (−1.104 to 0.128) | 0.509 | Adjusted for interaction %WL ≥5%*sex |

β, beta coefficients; CI, confidence interval; %WL, percentage of weight loss; ΔWC, change in waist circumference; AST, aspartate aminotransferase; ALT, alanine aminotransferase; GGT, gamma-glutamyl transferase; LDL, low-density lipoprotein cholesterol; HSI, hepatic steatosis index; FLI, fatty liver index; FIB-4, fibrosis-4 index; APRI, AST-to-platelet ratio index; VAI, visceral adiposity index.

**Table S6.** Baseline anthropometric characteristics and semaglutide dose according to prior GLP-1 RAs exposure (naïve vs switchers).

|  | Naïve | Switchers | P-value |
| --- | --- | --- | --- |
| Weight (kg) | 95.2 (96.9-102.2) | 92.2 (90.2-99.2) | 0.126 |
| BMI (kg/m^2^) | 35.9 (35.5-37.1) | 33.1 (33.3-36.1) | 0.060 |
| WC (cm) | 108.0 (107.8-112.6) | 107.0 (103.3-111.5) | 0.452 |
| WHtR | 0.67 (0.58-0.66) | 0.65 (0.62-0.67) | 0.475 |
| Semaglutide dose T1-T0 (mg) | 0.75 (0.7, 0.8) | 0.95 (0.8, 1.1) | **0.003** |
| Semaglutide dose T2-T0 (mg) | 1.2 (1.2, 1.4) | 1.2 (1.0, 1.5) | 0.488 |
| Semaglutide dose T3-T0 (mg) | 1.0 (1.0, 1.4) | 1.2 (1.0, 1.7) | 0.806 |

Data are expressed as median and IQR (25-75). BMI, Body Mass Index; WC, Waist Circumference; WHtR, Waist to Height Ratio.

**Table S7.** Changes in anthropometric parameters after treatment according to sex.

|  | Women | Δ% | Men | Δ% | p-value |
| --- | --- | --- | --- | --- | --- |
| Weight  (kg) |  |  |  |  |  |
| T1-T0 | -5.1 (-6.2, -4.9)  N=156 | -5.5 (-6.7, -5.4) | -5.0 (-6.9, -3.8)  N=37 | -4.7 (-6.0, -3.6) | 0.606 |
| T2-T0 | -9.1 (-10.7,-8.7)  N=125 | -9.9 (-15.9,-8.6) | -10.4 (-15.0,-9.0)  N= 28 | -9.4 (-12.0, -8.5) | 0.138 |
| T3-T0 | -12.0 (-15.7,-11.5)  N=48 | -15.2 (-17.8,-13.6) | -13.8 (-20.3,-7.8)  N=7 | -12.6 (-14.0,-10.8) | 0.665 |
| BMI  (kg/m^2^) |  |  |  |  |  |
| T1-T0 | -2.0 (-2.3, -1.9)  N=156 |  | -1.7 (-2.3, -1.3)  N=37 |  | 0.221 |
| T2-T0 | -3.6 (-4.9,-3.2)  N=125 |  | -3.5 (-4.8, -3.1)  N= 28 |  | 0.745 |
| T3-T0 | -4.5 (-6.8,-4.4)  N=48 |  | -4.7 (-6.8,-2.5)  N=7 |  | 0.753 |
| WC  (cm) |  |  |  |  |  |
| T1-T0 | -4.0 (-6.5,-4.4)  N= 87 |  | -5.0 (-8.7, -2.6)  N= 15 |  | 0.928 |
| T2-T0 | -9.0 (-11.0,-8.2)  N=68 |  | -13.0 (-18.9, -7.6)  N=9 |  | 0.146 |
| T3-T0 | -12.0 (-15.9,-10.4)  N=31 |  | -14.0 (-29.8,-1.5)  N=3 |  | 0.564 |
| WHtR |  |  |  |  |  |
| T1-T0 | -0.026 (-0.04,-0.03)  N= 87 |  | -0.031 (-0.07,-0.02)  N= 15 |  | 0.661 |
| T2-T0 | -0.054 (-0.07,-0.05)  N=68 |  | -0.073 (-0.10, 0.04)  N=9 |  | 0.333 |
| T3-T0 | -0.076 (-0.09,-0.04)  N=31 |  | -0.078 (-0.17,-0.05)  N=3 |  | 0.731 |

Data are expressed as median and IQR (25-75). BMI, Body Mass Index; WC, Waist Circumference; WHtR, Waist to Height Ratio; WL, Weight Loss.

**Table S8.** Changes in anthropometric parameters after treatment according to baseline BMI categories.

| BMI  categories (kg/m^2^) | 25-29.99 | 30-34.99 | 35-39.99 | ≥40 | p-value |
| --- | --- | --- | --- | --- | --- |
| Weight  (kg) |  |  |  |  |  |
| T1-T0 | -5.0 (-8.0, -3.8)  N=27 | -5.5 (-6.0,-4.4)  N=71 | -5.5 (-7.0,-4.6)  N=48 | -5.0 (-7.1,-4.4)  N=45 | 0.504 |
| T2-T0 | -8.5 (-10.7,-7.2)  N=21 | -9.5 (-10.5,-8.1)  N= 59 | -9.0 (-12.0,-8.4)  N=39 | -11.0 (-15.7,-9.6)  N=33 | 0.776 |
| T3-T0 | -11.2 (-14.1,-4.7)  N=6 | -11.4 (-14.6,-10.6)  N=21 | -13.8 (-20.7, -11.2)  N=15 | -17.7 (-21.0, -11.2)  N=12 | 0.100 |
| BMI  (kg/m^2^) |  |  |  |  |  |
| T1-T0 | -1.87 (-2.7, -1.4)  N=27 | -2.0 (-2.2, -1.6)  N=71 | -1.95 (-2.5,-1.7)  N=48 | -1.80 (-2.7,-1.7)  N=45 | 0.703 |
| T2-T0 | -3.3 (-4.0, -2.7)  N=21 | -3.7 (-3.8, -2.9)  N= 59 | -3.2 (-4.4,-3.0)  N=39 | -4.6 (-8.9,-3.1)  N=33 | 0.956 |
| T3-T0 | -4.2 (-15.8,1.7)  N=6 | -4.4 (-5.5,-4.0)  N=21 | -4.7 (-8.0,-4.2)  N=15 | -6.1 (-7.6,-4.0)  N=12 | 0.212 |
| WC  (cm) |  |  |  |  |  |
| T1-T0 | -6.0 (-10.2,-4.1)  N= 17 | -4.0 (-6.5, -3.6)  N= 40 | -3.5 (-5.6,-3.2)  N=26 | -4.0 (-10.1, -3.0)  N=18 | 0.069 |
| T2-T0 | -10.0 (-13.0,-7.1)  N= 13 | -9.0 (-12.2, -8.1)  N= 31 | -8.5 (-10.7,-5.8)  N=19 | -9.0 (-17.3,-7.1)  N=14 | 0.612 |
| T3-T0 | -12.0 (-20.2,-5.1)  N=3 | -14.0 (-17.8,-11.2)  N=13 | -11.0 (-18.6,-6.8)  N=11 | -14.0 (-22.0,-9.3)  N=6 | 0.333 |
| WHtR |  |  |  |  |  |
| T1-T0 | -0.037 (-0.06,-0.02)  N= 17 | -0.025 (-0.04,-0.02)  N= 40 | -0.022 (-0.03,-0.02)  N=26 | -0.032 (-0.08,-0.02)  N=18 | 0.101 |
| T2-T0 | -0.056 (-0.08,-0.04)  N= 13 | -0.058 (-0.08, 0.05)  N= 31 | -0.051 (-0.06,-0.03)  N=19 | -0.056 (-0.10,-0.04)  N=14 | 0.519 |
| T3-T0 | -0.076 (-0.12,-0.04)  N=3 | -0.093 (-0.11,-0.05)  N=13 | -0.061 (-0.13,0.03)  N=11 | -0.087 (-0.13,-0.06)  N=6 | 0.446 |
| WL  (%) |  |  |  |  |  |
| T1-T0 | -6.5 (-9.8, -5.1)  N=27 | -6.2 (-6.8,-5.1)  N=71 | -5.2 (-7.0,-4.7)  N=48 | -4.1 (-5.8,-3.7)  N=45 | 0.305 |
| T2-T0 | -10.9 (-13.7,-9.1)  N=21 | -11.0 (-11.7, -9.1)  N= 59 | -8.3 (-12.0,-8.1)  N=39 | -9.5 (-12.4,-8.1)  N=33 | 0.472 |
| T3-T0 | -14.3 (-16.2,-11.9)  N=6 | -12.9 (-17.0,-11.6)  N=21 | -18.0 (-23.4,-13.3)  N=15 | -14.0 (-16.7,-11.5)  N=12 | 0.729 |

Data are expressed as median and IQR (25-75). BMI, Body Mass Index; WC, Waist Circumference; WHtR, Waist to Height Ratio; WL, Weight Loss.

## 1.2 Supplementary Figures

**Figure S1.** Percentage achieving 5%, 10%, and 15% weight loss according to prior liraglutide exposure (naïve vs switchers). WL, Weight Loss.

**Figure S2.** Percentage achieving 5%, 10%, and 15% weight loss by sex. WL, Weight Loss.
